# ADAPTING A PHYSICAL ACTIVITY INTERVENTION FOR RURAL AREAS: PEOPLE WITH MCI AND CARE PARTNER DYADS

**DOI:** 10.1093/geroni/igad104.1318

**Published:** 2023-12-21

**Authors:** Rashelle Hoffman, Shinobu Watanabe-Galloway, Catherine Riffin, Joan Monin

**Affiliations:** Creighton University, Omaha, Nebraska, United States; University of Nebraska Medical Center, Omaha, Nebraska, United States; Weill Cornell Medicine, New York City, New York, United States; Yale School of Public Health, New Haven, Connecticut, United States

## Abstract

Rural populations face an increased risk of Alzheimer’s Disease (AD) compared to urban populations. Physical inactivity is a modifiable risk factor of AD and a prevalent issue for people with mild cognitive impairment (MCI) that can be addressed remotely with telerehabilitation. However, rural older adults differ from urban-dwelling as they often view physical activity as physical labor and have unique barriers (e.g., low social support). Involving a care partner for social support and improving physical activity is essential for people with MCI. The telerehabilitation physical activity behavioral (TPAB) intervention involves weekly discussions about self-monitoring physical activity with a FitBit, setting goals, and discussing personal barriers/facilitators and has been shown to be feasible and efficacious to increase daily step count in other older adult populations. Evidence is lacking for the TPAB’s use in people with MCI living in rural settings and their care partners (patient + care partner dyads). Results from the first phase of our study include three focus groups (four people per group) from a Nebraska rural community including medical providers, care partners, and older community-dwelling adults to identify ways to adapt the study design and TPAB intervention for rural patient + care partner dyad implementation. Discussion will focus on feasibility (Person-Based Approach) and adaptation (FRAME framework) of the TPAB intervention for implementation in rural communities. The results of this project will lead towards using telehealth technology to overcome detrimental geographical impacts on health (i.e., living in rural communities) to advance the care for patient + care partner dyads.

